# Incidence of Hepatitis C Virus (HCV) in a Multicenter Cohort of HIV-Positive Patients in Spain 2004–2011: Increasing Rates of HCV Diagnosis but Not of HCV Seroconversions

**DOI:** 10.1371/journal.pone.0116226

**Published:** 2014-12-30

**Authors:** Paz Sobrino-Vegas, Susana Monge Corella, Sergio Serrano-Villar, Félix Gutiérrez, José Ramón Blanco, Ignacio Santos, Jorge del Romero, Ferrán Segura, Joaquín Portilla, Santiago Moreno Guillén, Julia del Amo, CoRIS

**Affiliations:** 1 Red de Investigación en Sida, Centro Nacional de Epidemiología, Instituto de Salud Carlos III, Madrid, Spain; 2 Hospital Universitario Ramón y Cajal, Madrid, Spain; 3 Hospital General Universitario de Elche, Elche, Spain; 4 Hospital San Pedro, Logroño, Spain; 5 Hospital Universitario de La Princesa, Madrid, Spain; 6 Centro Sanitario Sandoval, Madrid, Spain; 7 Hospital Universitari Parc Taulí, Sabadell, Spain; 8 Hospital General de Alicante, Alicante, Spain; 9 Cohorte de la Red de Investigación en Sida, Madrid, Spain; Kaohsiung Medical University Hospital, Kaohsiung Medical University, Taiwan

## Abstract

**Objectives:**

We aim to describe rates and risk factors of Hepatitis C Virus (HCV) diagnoses, follow-up HCV testing and HCV seroconversion from 2004–2011 in a cohort of HIV-positive persons in Spain.

**Methods:**

CoRIS is a multicentre, open and prospective cohort recruiting adult HIV-positive patients naïve to antiretroviral therapy. We analysed patients with at least one negative and one follow-up HCV serology. Incidence Rates (IR) were calculated and multivariate Poisson regression was used to estimate adjusted Rates Ratios (aIRR).

**Results:**

Of 2112 subjects, 53 HCV diagnoses were observed, IR = 0.93/100py (95%CI: 0.7–1.2). IR increased from 0.88 in 2004–05 to 1.36 in 2010–11 (aIRR = 1.55; 95%CI: 0.37–6.55). In men who have sex with men (MSM) from 0.76 to 1.10 (aIRR = 1.45; 95%CI: 0.31–6.82); in heterosexual (HTX) subjects from 1.19 to 1.28 (aIRR = 1.08; 95%CI: 0.11–10.24). HCV seroconversion rates decreased from 1.77 to 0.65 (aIRR = 0.37; 95%CI: 0.12–1.11); in MSM from 1.06 to 0.49 (aIRR = 0.46; 95%CI: 0.09–2.31); in HTX from 2.55 to 0.59 (aIRR = 0.23; 95%CI: 0.06–0.98). HCV infection risk was higher for injecting drug users (IDU) compared to HTX (aIRR = 9.63;95%CI: 2.9–32.2); among MSM, for subjects aged 40–50 compared to 30 or less (IRR = 3.21; 95%CI: 1.7–6.2); and among HTX, for female sex (aIRR = 2.35; 95%CI: 1.03–5.34) and <200 CD4-count (aIRR = 2.39; 95%CI: 0.83–6.89).

**Conclusion:**

We report increases in HCV diagnoses rates which seem secondary to intensification of HCV follow-up testing but not to rises in HCV infection rates. HCV IR is higher in IDU. In MSM, HCV IR increases with age. Among HTX, HCV IR is higher in women and in subjects with impaired immunological situation.

## Introduction

The epidemiology of hepatitis C virus (HCV) infection in persons living with human immunodeficiency virus (HIV) in Western countries has changed profoundly in the last 15 years [1]–[12]. Outbreaks of acute HCV infection have been reported in HIV-positive men who have sex with men (MSM) from the mid-2000s onwards in large cities in Europe [3]–[7], [9], [13] and the United States (US) [1] and decreases in the numbers of injecting drug users (IDU) in industrialized countries have led to decreases in HCV serial prevalence [14]–[16].

The impact of these HCV outbreaks on the global epidemiology of HCV infection, particularly among HIV-positive patients, is yet to be described. In spite of the relevance of HCV co-infection [17], surveillance of HCV is not sufficiently developed in most European countries. Several European cohort studies have described increasing rates of HCV diagnoses as well as of HCV seroconversions in MSM since the mid-eighties to current date, but more markedly from 2002 onwards [2], [8], [10]–[11]. The Multicentre AIDS Cohort Study (MACS) has reported the on-going HCV epidemic in HIV-positive MSM in the US from 1984 to 2011 but describes no actual increases of HCV infection over time [12].

Early diagnosis of HCV infection is the first step to access treatment, HCV eradication and decreased on-going HCV transmission. However, though HCV testing is recommended to all HIV-positive persons, there are currently no guidelines on how often should HCV testing be repeated in HIV-positive patients in the absence of clinical symptoms.

The objective of this work is to describe the rates and risk factors of HCV diagnoses, follow-up HCV testing and HCV seroconversion from 2004 to 2011, in a multicentre cohort of HIV-positive persons in Spain. We show the results globally, for MSM and for people infected through heterosexual transmission (HTX).

## Methods

CoRIS (Spanish acronym for the AIDS Research Network Cohort) is an open, prospective, multicentre cohort of adult (age 18 or older) patients with confirmed HIV infection. It includes patients who began follow-up in any of the participating sites after January 2004 while naïve to antiretroviral treatment (cART).

Ethics approval was obtained from all hospitals Ethics’ Committees (see Annex for all hospitals participanting) and every patient provides a written informed consent to participate in the cohort.

A complete description of the cohort has been published elsewhere [18]. Currently, 31 groups from 28 public health-care centres within 13 of Spain’s 17 Autonomous Regions participate in the cohort. Patients' follow-up and frequency of HCV testing are established by the physician in charge as there are no standard recommendations for follow-up HCV testing in HIV-positive persons in Spain.

HIV-positive subjects were recruited from 2004 onwards up to the 31^st^ October 2011– administrative censoring date. Eligibility criteria for these analyses required having a documented negative HCV serology before or upon cohort recruitment and at least one follow-up HCV test performed. Since eligibility for a follow-up HCV test also depends on the length of clinical follow-up and the number of visits, we further excluded patients with clinical follow-up periods under 2 years.

### Definition of study outcomes

HCV diagnosis was defined based on positive HCV antibodies using a third generation EIA (enzyme immunoassay). HCV diagnoses were verified by consecutive confirmatory HCV-positive serologies, positive antigen determinations and/or HCV viral load detection. HCV diagnoses that could not be confirmed were also included, but sensitivity analyses excluding these cases were also performed.

The date of HCV diagnosis was the date the first HCV test was positive. Incidence rates of HCV diagnosis were calculated as the number of HCV diagnoses per 100 person-years at risk (p-y), and patients were considered at risk from the date of cohort entry to the date of their last negative HCV serology or the date of HCV diagnosis.

The date of HCV infection (or date of HCV seroconversion) was estimated using two different methods: a) the midpoint between the last negative and the first positive HCV test for any time window (*midpoint method*) and b) a multiple imputation technique (*multiple imputation method).* Rates of HCV infection were calculated as the number of HCV seroconversions - excluding those estimated before the date of patient’s inclusion in the cohort - per 100 person-years at risk (p-y), and patients were considered at risk from the date of cohort entry to the date of their last negative HCV serology or the date of HCV infection.

Rate of follow-up HCV tests were calculated as the number of persons with follow-up HCV test in each period, per 100 person-years at risk of HCV diagnosis.

### Statistical analyses

Logistic regression models were fitted to evaluate sociodemographic characteristics associated with a follow-up HCV test (gender, age at cohort entry, educational level, country of origin, and transmission category -in mutually excluding categories and prioritizing those with a higher risk of transmission-).

The multiple imputation technique was performed as follows: for each diagnosis, the HCV seroconversion date (estimated date of infection) was imputed by extracting a random date from a uniform distribution between the last negative and the first positive HCV test, assuming that seroconversion was equally probable at any given moment during that time window. This process was replicated 20 times for each subject and a separate database was generated for each replica. The final estimates were the result of combining individual estimates from each replica using the procedure described by Rubin, which accounts for the observed variability between estimations in the estimation of the global variance [19].

Multivariate Poisson regression models were fitted to evaluate a priori established sociodemographic risk factors for HCV incidence and CD4 cell count, HIV Viral Load and cART, as time-changing variables.

To analyse trends in HCV incidence, Poisson regression models were used, evaluating the presence of confounding variables for the association between calendar period and HCV incidence. We obtained crude (IRR) and adjusted (aIRR) incidence rate ratios, and their confidence intervals at 95% (95%CI).

We performed various simulations to evaluate the potential source of selection bias derived from the exclusion of subjects who had not been tested for HCV infection during follow-up assuming they had a different risk of HCV infection. We assumed all subjects with a minimum of two years of follow-up had been tested for HCV infection on the date of their last clinic visit and calculated the number of simulated HCV infections for each calendar year under two different scenarios assuming high and arbitrary HCV incidence rates; (1) a constant HCV rate of 1.5 per 100 p-y, and (2) an increasing HCV rate overtime starting at 1.5 per 100 p-y in years 2004.05 mounting to 2.00 per 100 p-y in 2010–11. We aggregated these numbers to the main analyses and obtained HCV time trends under these simulated scenarios.

Finally, we conducted sensitivity analyses excluding the information of all HCV serologies performed before cohort entry; that is, limiting analyses to subjects with at least 2 HCV tests from cohort recruitment onwards.

Robust methods were used to estimate confidence intervals assuming a correlation between subjects recruited at each site, and independence between subjects recruited at different sites [20]. Analyses were conducted using Stata (V.11, Stata Corporation, College Station, Texas, USA).

## Results

**Figure 1:**
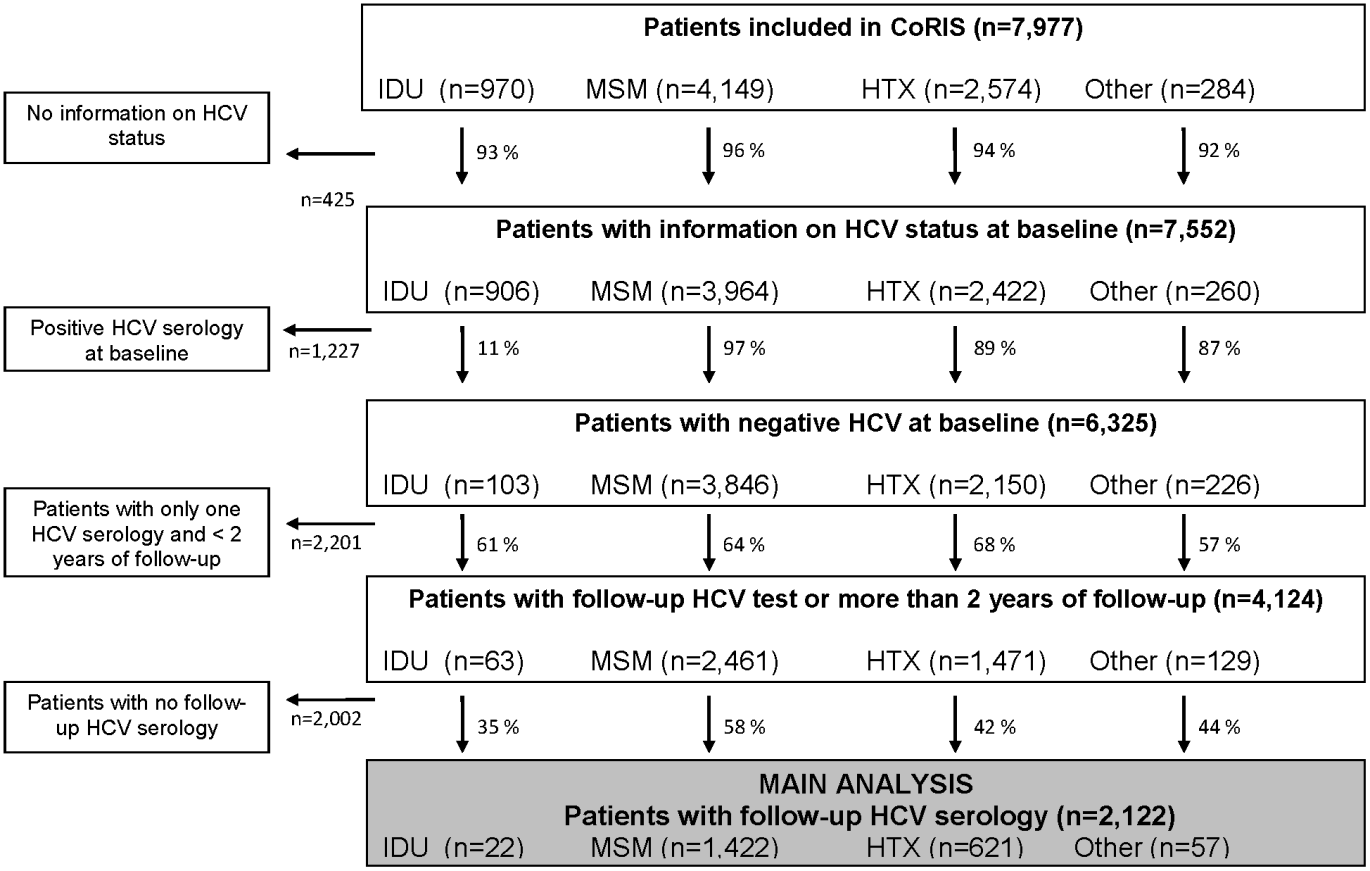
Patients flow-chart.

**Figure 2:**
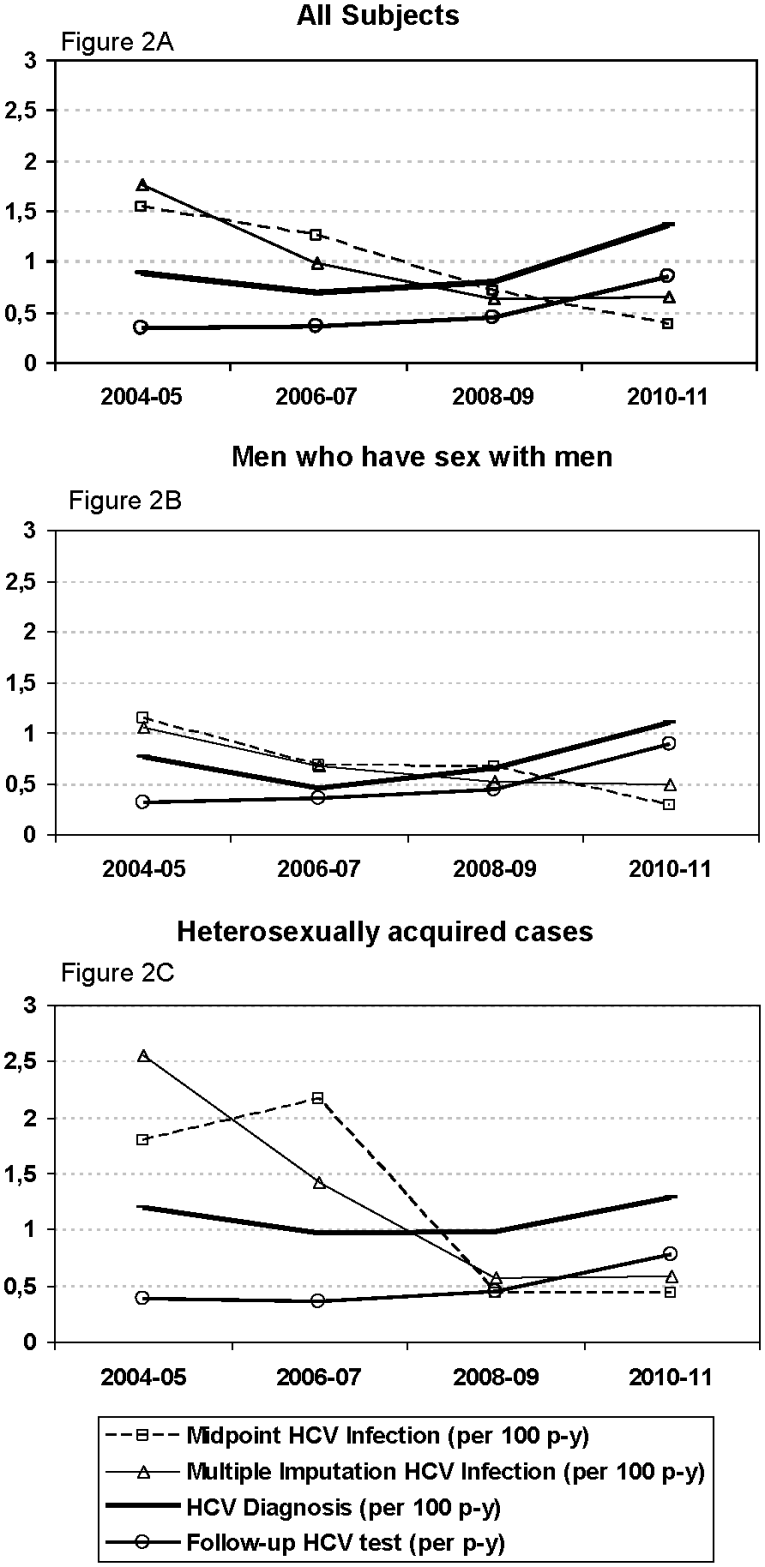
Time trends of rates of HCV diagnoses, HCV infections and of follow-up HCV tests, 2004–2011.

Out of 7977 patients, baseline HCV serology was available for 7552 (Fig. 1). At cohort recruitment, 1227 had a positive HCV serology; prevalent cases were older and mainly IDU or ex-IDU (65%). Of the remaining 6425 patients with negative baseline HCV serology, 4124 had at least one follow-up HCV test or more than two years of follow-up (Fig. 1). In all, 2122 patients had at least one HCV test repeated over time. Median time between HCV serologies was 1.34 years (IQR: 0.43–2.49) and median number of serologies per patient was 2 (IQR: 2–3). Follow-up HCV testing was commoner in MSM, people under 50 years of age and non-Spanish origin (Table 1). Further, the rate of follow-up tests increased over time, reaching up to 85 follow-up HCV test per 100 person-years in the period 2010–2011; 89 per 100 p-y for MSM and 78 per 100 p-y for the heterosexually infected (Table 2 and Fig. 2A, 2B, 2C). The percentage of positive HCV diagnoses among the tested individuals dropped slightly over this period (2.6% in 2004–05 to 1.6% in 2010–11).

**Table 1:** Sociodemographic characteristics of 4124 patients with follow-up HCVtest or more than two years of follow-up: prevalence of repeated HCV tests and multivariable logistic regression for the odds ratio (OR) of having a subsequent HCV serology.

	Patients with follow-up HCV test or more than two years of follow-up (n = 4,124)						Adjusted OR (95% CI)
	n	%	Patients with follow-up HCV serology (n = 2,122)		Patients without follow-up HCV serology (n = 2,002)		
			n	%	n	%	
**SEX**							
Male	3356	81.4	1797	53.5	1559	46.5	1
Female	768	18.6	325	42.3	443	57.7	0.96 (0.78–1.17)
**TRANSMISSION CATEGORY**							
Heterosexuals	1471	35.7	621	42.2	850	57.8	1
Injecting drug users	63	1.5	22	34.9	41	65.1	0.74 (0.43–1.26)
Homo/bisexual men	2461	59.7	1422	57.8	1039	42.2	1.79 (1.50–2.13)
Other/Unknown/Not available	129	3.1	57	44.2	72	55.8	1.04 (0.72–1.51)
**AGE AT ENTRY**							
< = 30 years	1423	34.5	752	52.8	671	47.2	1.29 (1.02–1.65)
31–40 years	1581	38.3	843	53.3	738	46.7	1.36 (1.07–1.72)
41–50 years	745	18.1	370	49.7	375	50.3	1.27 (0.98–1.64)
>50 years	375	9.1	157	41.9	218	58.1	1
**EDUCATION LEVEL**							
Low	1301	31.5	627	48.2	674	51.8	1
Medium	1419	34.4	749	52.8	670	47.2	1.00 (0.85–1.17)
High	942	22.8	533	56.6	409	43.4	1.10 (0.92–1.31)
Not available	462	11.2	213	46.1	249	53.9	0.89 (0.72–1.10)
**COUNTRY OF ORIGIN**							
Spain	2794	67.7	1416	50.7	1378	49.3	1
Other	1317	31.9	694	52.7	623	47.3	1.17 (1.02–1.34)
Not available	13	.3	12		1		–
**TOTAL**	4124	100.0	2122	51.5	2002	48.5	

**Table 2:** Evolution of follow-up HCV testing rates, of HCV diagnosis rates and of HCV infection rates (per 100 person-years).

	All subjects													
	Follow-up HCVtests (n = 2,122)				HCV diagnoses(n = 2,122)			HCV Infections						
								MidpointMethod(n = 2,115)			MultipleImputationMethod (n = 2,121)			
	n	p-y	IR	IRR (95% CI)	n	IR	IRR (95% CI)	n	IR	IRR (95% CI)	n	IR	IRR (95% CI)	
**PERIOD**														
2004–05	154	455.50	34	1	4	0.88	1	7	1.54	1	8.10	1.77	1	
2006–07	513	1452.58	35	1.04 (0.87–1.25)	10	0.69	0.78 (0.30–2.05)	18	1.25	0.81 (0.39–1.66)	14.30	0.99	0.56 (0.22–1.43)	
2008–09	950	2162.46	44	1.30 (1.10–1.54)	17	0.79	0.90 (0.24–3.30)	15	0.70	0.45 (0.17–1.20)	13.80	0.64	0.36 (0.11–1.15)	
2010–11	1379	1618.40	85	2.52 (2.13–2.98)	22	1.36	1.55 (0.37–6.55)	6	0.37	0.24 (0.10–0.58)	10.50	0.65	0.37 (0.12–1.11)	
**TOTAL**	2996	5688.94	53		53	0.93		46	0.82		46.70	0.83		
	**Men who have sex with men**													
	**Follow-up HCV** **tests (n = 1,421)**				**HCV diagnoses** **(n = 1,421)**			**HCV Infections**						
								**Midpoint** **Method** **(n = 1,416)**			**Multiple** **Imputation** **Method (n = 1,421)**			
	**n**	**p-y**	**IR**	**IRR (95% CI)**	**n**	**IR**	**IRR (95% CI)**	**n**	**IR**	**IRR (95% CI)**	**n**	**IR**	**IRR (95% CI)**	
**PERIOD**														
2004–05	83	263.70	31	1	2	0.76	1	3	1.14	1	2.90	1.06	1	
2006–07	307	885.74	35	1.10 (0.86–1.40)	4	0.45	0.60 (0.17–2.07)	6	0.68	0.60 (0.18–1.96)	6.10	0.68	0.65 (0.13–3.23)	
2008–09	597	1374.98	43	1.38 (1.10–1.74)	9	0.65	0.86 (0.26–2.88)	9	0.66	0.58 (0.25–1.33)	7.35	0.52	0.50 (0.12–2.14)	
2010–11	966	1090,28	89	2.81 (2.25–3.52)	12	1.10	1.45 (0.31–6.82)	3	0.28	0.24 (0.06–0.90)	5.40	0.49	0.46 (0.09–2.31)	
**TOTAL**	1953	3614.70	54		27	0.75		21	0.58		21.75	0.60		
	**Heterosexually acquired cases**													
	**Follow-up HCV** **tests (n = 621)**				**HCV diagnoses** **(n = 621)**			**HCV Infections**						
								**Midpoint** **Method** **(n = 620)**			**Multiple** **Imputation** **Method (n = 621)**			
	**n**	**p-y**	**IR**	**IRR (95% CI)**	**n**	**IR**	**IRR (95% CI)**	**n**	**IR**	**IRR (95% CI)**	**n**	**IR**		**IRR (95% CI)**
**PERIOD**														
2004–05	64	168.60	38	1	2	1.19	1	3	1.79	1	4.40	2.55		1
2006–07	185	515.81	36	0.94 (0.71–1.26)	5	0.97	0.82 (0.14–4.78)	11	2.16	1.21 (0.27–5.38)	7.45	1.43		0.56 (0.12–2.63)
2008–09	319	715.35	45	1.17 (0.90–1.54)	7	0.98	0.82 (0.08–8.11)	3	0.43	0.24 (0.03–1.75)	4.20	0.57		0.22 (0.03–1.43)
2010–11	365	470.40	78	2.04 (1.57–2.67)	6	1.28	1.08 (0.11–0.24)	2	0.43	0.24 (0.10–0.58)	2.90	0.59		0.23 (0.06–0.98)
**TOTAL**	933	1870.15	50		20	1.07		19	1.03		18.95	1.03		

The majority of these 2122 persons were men (85%), infected through sexual transmission (67% MSM and 29.3% HTX), and of Spanish origin (67%). Overall, 35% were under 30 and 25% over 50, with ages between 18 and 77 (Table 1). Median CD4 count at recruitment was 399 (IQR: 224–592), median viral load was 4.6 log (IQR: 4.06–5.15) and 76% started cART during follow-up.

These 2122 individuals added up to 5689 p-y in whom 53 HCV diagnoses were observed. In 48 cases, diagnosis could be confirmed and in five no further serologic determinations had been performed. HCV diagnosis rate was IR = 0.93 (95%CI: 0.71–1.22) (Table 2). Global HCV diagnosis rates increased from 0.88 in 2004–05 to 1.36 in 2010–11 (IRR 1.55; 95%CI: 0.37–6.55) (Table 2, Fig. 2A). Among MSM, rates of HCV diagnosis increased from 0.76 to 1.10 per 100 p-y (IRR 1.45; 95%CI: 0.31–6.82) (Table 2, Fig. 2B) and in HTX from 1.19 to 1.28 per 100 p-y (IRR 1.08; 95%CI: 0.11–10.24) (Table 2, Fig. 2C).

Using the multiple imputation method, infection rate was 0.83 (95%CI: 0.61–1.13) after excluding the one individual with all imputed seroconversion dates before cohort entry. HCV infection rate decreased from 1.77 to 0.65 per 100 p-y (IRR = 0.37; 95%CI: 0.12–1.11) (Table 2, Fig. 2A). Among MSM it decreased from 1.06 to 0.49 per 100 p-y (IRR 0.46; 95%CI: 0.09–2.31) (Table 2, Fig. 2B). In HTX it decreased from 2.55 to 0.59 per 100 p-y (IRR 0.23; 95%CI: 0.06–0.98) (Table 2, Fig. 2C). Using the midpoint method, infection rate was 0.82 per 100 p-y (95%CI: 0.61–1.09) -seven patients were excluded because their seroconversion date was before cohort entry-. Time trends of HCV infection rate using this method are showed in Table 2 and Fig. 2. Only unadjusted results are shown, as they were not confounded by any of the variables studied.

Factors independently associated with higher HCV infection were: being an IDU (aIRR 9.63; 95%CI: 2.88–32.19) and, though not reaching statistical significance, age 41–50 (aIRR 1.66; 95%CI: 0.85–3.24) and CD4 cell count below 200 cells/mm^3^ (aIRR 1.93; 95%CI: 0.90–4.15) (Table 3). In analyses restricted to MSM, the only factor associated with a higher HCV infection was age 41–50 (IRR 3.21; 95%CI: 1.66–6.23). Among HTX, two factors showed independent association with higher HCV infection rate: female sex (aIRR 2.35; 95%CI: 1.03–5.34) and CD4 cell count below 200 cells/mm^3^ (aIRR 2.39; 95%CI: 0.83–6.89).

**Table 3:** Rates and associated risk factors of HCV infection.

	ALL subjects (n = 2,115)				
	Infections	Person-years	IR	IRR (CI 95%)	aIRR (CI 95%)
**SEX**					
Male	33	4694.28	0.70	1	1
Female	13	941.43	1.38	1.96 (1.03–3.76)	1.58 (0.78–3.20)
**TRANSMISSION CATEGORY**					
Heterosexuals	19	1846.42	1.03	1	1
Injecting drug users	5	53.31	9.38	9.11 (2.86–29.08)	9.63 (2.88–32.19)
Homo/bisexual men	21	3592.28	0.58	0.57 (0.24–1.36)	0.81 (0.29–2.27)
Other/Unknown	1	143.70	0.70	0.68 (0.10–4.79)	0.77 (0.11–5.50)
**AGE AT ENTRY**					
< = 30 years	11	1866.73	0.59	1	1
31–40 years	19	2350.63	0.81	1.37 (0.57–3.30)	1.38 (0.57–3.30)
41–50 years	12	960.44	1.25	2.12 (1.15–3.90)	1.66 (0.85–3.24)
>50 years	4	457.91	0.87	1.48 (0.68–3.24)	1.29 (0.53–3.14)
**CD4+ T-Cell Count**					
<200 cells/mm^3^	8	455.76	1.76	2.36 (1.17–4.75)	1.93 (0.90–4.15)
> = 200 cells/mm^3^	38	5100.96	0.74	1	1
Not available	0	79.00	0.00		
**TOTAL**	46	5635.72	0.82		
	**Men who have sex with men (n = 1,416)**				
	**Infections**	**Person-years**	**IR**	**IRR (CI 95%)**	**aIRR (CI 95%)**
**AGE AT ENTRY**					
< = 30 years	5	1314.77	0.38	1	1
31–40 years	9	1562.66	0.58	1.51 (0.44–5.22)	1.51 (0.44–5.22)
41–50 years	6	490.79	1.22	3.21 (1.66–6.23)	3.21 (1.66–6.23)
>50 years	1	224.06	0.45	1.17 (0.24–5.67)	1.17 (0.24–5.67)
**TOTAL**	21	3592.28	0.58		
	**Heterosexually acquired cases (n = 620)**				
	**Infections**	**Person-years**	**IR**	**IRR (CI 95%)**	**aIRR (CI 95%)**
**SEX**					
Male	6	943.48	0.64	1	1
Female	13	902.95	1.44	2.26 (1.01–5.05)	2.35 (1.03–5.34)
**CD4+ T-Cell Count**					
<200 cells/mm^3^	5	247.88	2.02	2.26 (0.78–6.53)	2.39 (0.83–6.89)
> = 200 cells/mm^3^	14	1569.37	0.89	1	1
Not available	0	29.17	0.00	–-	–-
**TOTAL**	19	1846.42	1.03		

*The table shows all the variables included in the final multivariables models.

The analyses of the associated risk factors yielded similar results using the two methods to estimate HCV seroconversion, for the cohort as a whole and for the two transmission categories analysed separately (Table 4 in S1 File).

### Results from simulation and sensitivity analyses

The simulation analysis assuming a constant HCV infection rate at 1.5 per 100 p-y throughout the study period among patients without serological follow-up, still showed a decrease in global infections rates. Assuming an increasing trend in HCV infection, from 1.5 in 2004/05 to 2.0 per 100 p-y in 2010/11, did not led to increases in HCV global infections rates, either (Tables 5a–5b in S2 File).

Excluding the HCV serologies performed before cohort entry restricted the sample to 1490 patients and 4003 p-y of serological follow-up. Sensitivity analyses showed very similar results for the incidence rate and risk factors, as well as for the time-trend analyses, although some were underpowered (Tables 6–7 and Figure 3 in S3 File). Excluding non-confirmed HCV diagnoses did not change findings either (Tables 8–9 and Figure 4 in S4 File).

## Discussion

The HCV infection rate observed in our cohort is 0.82 per 100 p-y globally, 9.4 per 100 p-y in IDU, 0.6 per 100 p-y in MSM and heterosexually infected men, and 1.4 per 100 p-y in heterosexually infected women. We report increases in the rates of HCV diagnoses in the last years which seem secondary to intensification of HCV follow-up testing but not to rises in HCV infection rates in a cohort of HIV-positive patients between 2004 and 2011 in Spain.

Injecting drug use remains the strongest risk factor for HCV infection rates, though the number of IDUs is now very low in the cohort. MSM aged 41–50, female heterosexuals and heterosexuals with CD4 cell count below 200 cells/mm^3^ had increased HCV infection rates.

HCV infection rates are, similar to those reported by earlier works conducted in HIV positive individuals [2], [8], [12], [21], [22]. Eurosida reported global IR of 0.79 per 100 p-y during 2002–2010 [2]. In HIV-positive MSM, Van de Laart et al. reported IR of 0.87 per 100 p-y during 2000–2003 [8], Taylor et al. IR = 0.51 per 100 p-y during 1996–2008 [22], Gamage et al. IR = 0.6 per 100 p-y during 2002–2010 [21] and Witt et al. reported IR = 0.52 per 100 p-y during 2005–2011 [12].

The risk factors associated with HCV infection identified in these analysis are comparable to previous studies; HCV infection is far higher in IDU [8], [12], [21], [22], [23], [24] and, within the MSM, incidence increases with age [21] although it stabilizes after age 50. HCV infection rates were similar between MSM and men who only reported sex with women. Information bias due to under disclosure of past injecting drug use cannot be ruled out.

We have observed that the HCV infection rate in subjects with CD4 cell counts below 200 cells/mm^3^ is twice higher that of people with better immunological situation, although results did not reach statistical significance. A recently published study from the MACS describes that, in individuals with <500 CD4 cells/mm^3^, HCV incidence was inversely associated with CD4 count [12], but previous publications did not report these findings [11], [22]. We need to take into account that the statistical power of this result is limited, as the person-time spent with CD4 cell counts below 200 cells/mm^3^ is short, and the rate of follow-up HCV test was higher in subjects with <200CD4 (RR = 1.3; 95%CI: 1.2–1.4).

Follow-up HCV testing was more common in MSM, people under 50 years of age and non-Spanish origin. It seems that qualitative and quantitative changes regarding HCV testing have taken place in Spain in the last years. As a consequence of the alert raised by the outbreaks of HCV infection in MSM, it seems physicians are testing this group more frequently, increasing the number of positive diagnoses as well, contributing to the denominator, and thus yielding lower seroconversion rates. Further, selection bias due to more frequent and selective HCV testing to a subset of HTX in whom a higher risk of infection is perceived may also have taken place, particularly in the first period before HCV testing become more widespread. This could be the case of the female sexual partners of HCV-positive IDU. Heterosexual women had an HCV infection rate twice higher than heterosexual men, similar to the PRIMO cohort and consistent with unprotected sexual intercourse with male IDU [25]. Unfortunately, the characteristics of the sexual partner/s of cohort members are not collected in CoRIS. The decreasing trend of HCV infection in CoRIS is concordant with the decrease in serial prevalence of HCV infection biomarkers reported by the Spanish national biosurveillance network [26], as well as with the serial HCV prevalence reported in CoRIS [14], [15].

Our time-trends results need to be placed in context of works which have found an increasing HCV incidence in European countries in recent years [2], some of them in MSM [8], [10], [11]. Eurosida reported an IR ranging from 0.47 to 2.34 per 100 p-y in the period 2002–2010 [2]. In HIV-positive MSM, Van de Laart et al. reported an IR varying from 0.08 to 0.87 per 100 p-y in 1984–2003 [8], the Swiss HIV Cohort an IR from 0.23 to 4.09 per 100 p-y in 1998–2011 [11], and in CASCADE Collaboration IR ranged depending on the method used from 1.6 and 3.0 in 2005 to 2.34 and 5.11 per 100 p-y in 2007 [10]. In HTX, the Swiss HIV Cohort reported a stabilized IR of less than 0.5 per 100 p-y [11].

For all groups and for MSM, we observe an increase of 50% in HCV diagnoses rates that did not reach statistical significance; the decrease in HCV infections was not significant either. This indicates that more cases are being diagnosed in recent years, but that does not mean more infections are necessarily occurring. In HTX, where the decrease in HCV seroconversions reaches statistical significance, the increased follow-up HCV testing does not lead to changes in the diagnosis rates.

For time-trends analysis we considered multiple imputation as the most appropriate method because the midpoint method underestimates the rates in the two extremes of the timeframe.

As a limitation, we acknowledge that HCV testing in our cohort was performed according to clinical criteria, rather than following a pre-specified screening scheme, so we cannot rule out that those with available follow-up serologies are those in which a higher probability of HCV infection was suspected; thus we could have overestimated the rates of HCV diagnoses and of HCV infection in our cohort. We think this is unlikely to having serious biased our results since the rate of patients that undergo serological follow-up has increased more than two-fold throughout the study period, to a similar degree in all transmission categories (34 to 86 per 100 p-y from 2004–05 to 2010–11), so it is possible that the non-significant increase we observe in HCV incidence is explained by an extension of serological follow-up to low-risk subjects.

Though CoRIS is not population-based, subjects included in the cohort show similar characteristics to those reported by Spanish national surveillance [27]. Therefore, our results can be extrapolated to the HIV-infected population in Spain. On the other hand, excluding patients’ serological information previous to cohort recruitment did not affect our estimates.

In conclusion, the increases in the rates of HCV diagnoses seem secondary to intensification of HCV follow-up testing, which is unveiling previously undiagnosed subjects, and not necessarily to rises in HCV infection. Repeating HCV tests to all HIV-positive persons under follow-up has yielded an increase in the number of cases, which is relevant both for clinical and public health purposes. Further follow-up of this cohort under similar HCV testing practices will provide additional evidence on the evolution of the rates of both HCV diagnosis and HCV infection in order to issue recommendations of HCV testing strategies in the HIV-positive population.

## Supporting Information

File S1 **Main Analysis: Results of multiple imputation method.** - Table 4. Rates and associated risk factors of HCV infection.
(DOC)

File S2 **Results of simulation analysis.** - Table 5a. Results obtained considering a constant HCV infection rate throughout the study period (IR = 1.5 per 100 p-y). - Table 5b. Results obtained considering an increasing HCV infection rate in the study period (IR from 1.5 to 2.0 per 100 p-y).
(DOC)

File S3 **Sensitivity analysis excluding the information of HCV serologies performed before cohort entry.** - Table 6. Evolution of HCV diagnosis rates and of HCV infection rates (per 100 p-y) - Table 7. Rates and associated risk factors of HCV infection. - Figure 3. Time trends of rates of HCV diagnoses, of HCV infections and of follow-up HCV tests, 2004–2011.
(DOC)

File S4 **Sensitivity analysis** **excluding HCV diagnoses that could not be confirmed.** - Table 8. Evolution of HCV diagnosis rates and of HCV infection rates (per 100 person-years). - Table 9. Rates and associated risk factors of HCV diagnoses and HCV Infections. - Figure 4. Time trends of rates of HCV diagnoses, of HCV infections and of follow-up HCV tests, 2004–2011.
(DOC)
