# Supplementary material for: Incidence of Hepatitis C Virus (HCV) in a Multicenter Cohort of HIV-Positive Patients in Spain 2004–2011: Increasing Rates of HCV Diagnosis but Not of HCV Seroconversions
Source: PLoS One. 2014 Dec 30;9(12):e116226. doi: 10.1371/journal.pone.0116226 (PMC4280214; doi:10.1371/journal.pone.0116226)
Supplement: File S1 — Main Analysis: Results of multiple imputation method. - Table 4. Rates and associated risk factors of HCV infection. (DOC) [file pone.0116226.s001.doc]

**MAIN ANALYSIS: RESULTS OF MULTIPLE IMPUTATIÓN METHOD**

94 %

92 %

89 %

87 %

29 %

25 %

74 %

**Table 4: Rates and associated risk factors of HCV infection**

92 %

87 %

25 %

74 %

94 %

92 %

89 %

87 %

29 %

25 %

74 %

|  | All subjects | | | | |
| --- | --- | --- | --- | --- | --- |
|  | **Infections** | **Person-years** | **IR** | **IRR (CI 95%)** | **aIRR (CI 95%)** |
| **SEX** |  |  |  |  |  |
| Male | 33.85 | 4696.08 | 0.72 | 1 | 1 |
| Female | 12.85 | 941.19 | 1.36 | 1.89 (0.98-3.67) | 1.54 (0.74-3.21) |
| **TRANSMISSIÓN** |  |  |  |  |  |
| Heterosexuals | 18.95 | 1846.96 | 1.03 | 1 | 1 |
| Injecting drug user | 5.00 | 53.40 | 9.37 | 9.14 (2.84-29.35) | 9.48 (2.81-31.96) |
| Homo/bisexual men | 21.75 | 3593.25 | 0.60 | 0.59 (0.25-1.38) | 0.83 (0.30-2.29) |
| Other /Unknown | 1.00 | 143.66 | 0.70 | 0.68 (0.10-4.80) | 0.77 (0.11-5.51) |
| **AGE AT ENTRY** |  |  |  |  |  |
| < =30 years | 11.50 | 1867.70 | 0.61 | 1 | 1 |
| 31-40 years | 18.75 | 2350.83 | 0.80 | 1.30 (0.55-3.07) | 1.29 (0.55-3.08) |
| 41-50 years | 12.45 | 960.91 | 1.29 | 2.11 (1.08-4.10) | 1.66 (0.79-3.48) |
| > 50 years | 4.00 | 457.84 | 0.87 | 1.42 (0.66-3.08) | 1.24 (0.51-3.01) |
| **CD4+ T-Cell Count** |  |  |  |  |  |
| <200 cel/mm^3^ | 8.15 | 455.59 | 1.77 | 2.36 (1.04-5.34) | 1.96 (0.83-4.65) |
| >= 200 cel/mm^3^ | 38.30 | 5102.57 | 0.75 | 1 | 1 |
| Not available | 0.25 | 79.11 | 1.77 | --- | --- |
| **TOTAL** | 46.70 | 5637.27 | 0.83 |  |  |
|  | **Men who have sex with men** | | | | |
|  | **Infections** | **Person-year** | **IR** | **IRR (CI 95%)** | **aIRR (CI 95%)** |
| **AGE AT ENTRY** |  |  |  |  |  |
| <=30 years | 5.55 | 1315.36 | 0.42 | 1 | 1 |
| 31-40 years | 8.80 | 1562.66 | 0.56 | 1.34 (0.39-4.62) | 1.34 (0.39-4.62) |
| 41-50 years | 6.40 | 491.18 | 1.30 | 3.11 (1.33-7.30) | 3.11 (1.33-7.30) |
| > 50 years | 1.00 | 224.05 | 0.45 | 1.07 (0.22-5.31) | 1.07 (0.22-5.31) |
| **TOTAL** | 21.75 | 3593.25 | 0.61 |  |  |
|  | **Heterosexually acquired cases** | | | | |
|  | **Infections** | **Person-year** | **IR** | **IRR (CI 95%)** | **aIRR (CI 95%)** |
| **SEX** |  |  |  |  |  |
| Male | 6.10 | 944.26 | 0.64 | 1 | 1 |
| Female | 12.85 | 902.70 | 1.42 | 2.21 (0.97-5.05) | 2.31 (0.98-5.43) |
| **CD4+ T-Cell Count** |  |  |  |  |  |
| <200 cel/mm^3^ | 5.60 | 248.00 | 2.23 | 2.63 (0.83-8.38) | 2.78 (0.87-8.89) |
| >= 200 cel/mm^3^ | 13.30 | 1569.79 | 0.85 | 1 | 1 |
| Not available | 0.05 | 29.17 | 0.00 | --- | --- |
| **TOTAL** | 18.95 | 1846.96 | 1.03 |  |  |
| (*) The table shows all the variables included in the final multivariables models | | | | | |
