# Supplementary material for: Incidence of Hepatitis C Virus (HCV) in a Multicenter Cohort of HIV-Positive Patients in Spain 2004–2011: Increasing Rates of HCV Diagnosis but Not of HCV Seroconversions
Source: PLoS One. 2014 Dec 30;9(12):e116226. doi: 10.1371/journal.pone.0116226 (PMC4280214; doi:10.1371/journal.pone.0116226)
Supplement: File S2 — Results of simulation analysis. - Table 5a. Results obtained considering a constant HCV infection rate throughout the study period (IR = 1.5 per 100 p-y). - Table 5b. Results obtained considering an increasing HCV infection rate in the study period (IR from 1.5 to 2.0 per 100 p-y). (DOC) [file pone.0116226.s002.doc]

RESULTS OF SIMULATION ANALYSIS

92 %

87 %

25 %

74 %

|  | **CoRIS** | | | | | | | | |
| --- | --- | --- | --- | --- | --- | --- | --- | --- | --- |
|  | **SIMULATION ANALYSIS**  **(n=2,002)** | | | **MAIN ANALYSIS**  **(n=2,122 )** | | | **GLOBALS RESULTS**  **(n=4,124 )** | | |
|  | **IR** | **Person-years** | **# Serocon-verters** | **Person-years** | **# Serocon-verters** | **IR** | **Person-years** | **# Serocon-verters** | **IR** |
| **PERIOD** |  |  |  |  |  |  |  |  |  |
| **2004-05** | **1.5** | 413.74 | 6 | 453.18 | 7 | 1.54 | 866.92 | 13 | **1.52** |
| **2006-07** | **1.5** | 1444.2 | 22 | 1441.42 | 18 | 1.25 | 2885.62 | 40 | **1.37** |
| **2008-09** | **1.5** | 2889.47 | 43 | 2137.82 | 15 | 0.70 | 5027.29 | 58 | **1.16** |
| **2010-11** | **1.5** | 3145.33 | 47 | 1603.30 | 6 | 0.37 | 4748.63 | 53 | **1.12** |
| **TOTAL** |  | 7892.74 | 118 | 5635.72 | 46 |  | 13528.46 | 164 |  |
|  | **CoRIS - HOMOSEXUAL/BISEXUAL MEN** | | | | | | | | |
|  | **SIMULATION ANALYSIS (n=1,039)** | | | **MAIN ANALYSIS**  **(n=1,422 )** | | | **GLOBALS RESULTS**  **(n=2,461 )** | | |
|  | **IR** | **Person-years** | **# Serocon-verters** | **Person-years** | **# Serocon-verters** | **IR** | **Person-years** | **# Serocon-verters** | **IR** |
| **PERIOD** |  |  |  |  |  |  |  |  |  |
| **2004-05** | **1.5** | 197.99 | 3 | 262.39 | 3 | 1.14 | 460.38 | 6 | **1.30** |
| **2006-07** | **1.5** | 653.47 | 10 | 881.71 | 6 | 0.68 | 1535.18 | 16 | **1.03** |
| **2008-09** | **1.5** | 1416.79 | 21 | 1364.55 | 9 | 0.66 | 2781.34 | 30 | **1.09** |
| **2010-11** | **1.5** | 1623.55 | 24 | 1083.63 | 3 | 0.28 | 2707.18 | 27 | **1.01** |
| **TOTAL** |  | 3891.79 | 58 | 3592.28 | 21 |  | 7484.07 | 79 |  |
|  | **CoRIS - HETEROSEXUALS** | | | | | | | | |
|  | **SIMULATION ANALYSIS**  **(n=850)** | | | **MAIN ANALYSIS**  **(n=621 )** | | | **GLOBALS RESULTS**  **(n=1,471 )** | | |
|  | **IR** | **Person-years** | **# Serocon-verters** | **Person-years** | **# Serocon-verters** | **IR** | **Person-years** | **# Serocon-verters** | **IR** |
| **PERIOD** |  |  |  |  |  |  |  |  |  |
| **2004-05** | **1.5** | 184.51 | 3 | 167.84 | 3 | 1.79 | 352.35 | 6 | **1.64** |
| **2006-07** | **1.5** | 694.84 | 10 | 509.45 | 11 | 2.16 | 1204.29 | 21 | **1.78** |
| **2008-09** | **1.5** | 1293.24 | 19 | 704.01 | 3 | 0.43 | 1997.25 | 22 | **1.12** |
| **2010-11** | **1.5** | 1340.08 | 20 | 465.13 | 2 | 0.43 | 1805.21 | 22 | **1.22** |
| **TOTAL** |  | 3512.67 | 53 | 1846.42 | 19 |  | 5359.09 | 72 |  |

**Table 5a: Results obtained considering a constant HCV infection rate throughout the study period (IR=1.5 per 100 p-y)**

**Table 5b: Results obtained considering an increasing HCV infection rate in the study period (IR from 1.5 to 2.0 per 100 p-y)**

|  | **CoRIS** | | | | | | | | |
| --- | --- | --- | --- | --- | --- | --- | --- | --- | --- |
|  | **SIMULATION ANALYSIS**  **(n=2,002)** | | | **MAIN ANALYSIS**  **(n=2,122 )** | | | **GLOBALS RESULTS**  **(n=4,124 )** | | |
|  | **IR** | **Person-years** | **# Serocon-verters** | **Person-years** | **# Serocon-verters** | **IR** | **Person-years** | **# Serocon-verters** | **IR** |
| **PERIOD** |  |  |  |  |  |  |  |  |  |
| **2004-05** | **1.50** | 413.74 | 6 | 453.18 | 7 | 1.54 | 866.92 | 13 | **1.52** |
| **2006-07** | **1.67** | 1444.2 | 24 | 1441.42 | 18 | 1.25 | 2885.62 | 42 | **1.46** |
| **2008-09** | **1.83** | 2889.47 | 53 | 2137.82 | 15 | 0.70 | 5027.29 | 68 | **1.35** |
| **2010-11** | **2.00** | 3145.33 | 63 | 1603.30 | 6 | 0.37 | 4748.63 | 69 | **1.45** |
| **TOTAL** |  | 7892.74 |  | 5635.72 | 46 |  | 13528.46 |  |  |
|  | **CoRIS - HOMOSEXUAL/BISEXUAL MEN** | | | | | | | | |
|  | **SIMULATION ANALYSIS (n=1,039)** | | | **MAIN ANALYSIS**  **(n=1,422 )** | | | **GLOBALS RESULTS**  **(n=2,461 )** | | |
|  | **IR** | **Person-years** | **# Serocon-verters** | **Person-years** | **# Serocon-verters** | **IR** | **Person-years** | **# Serocon-verters** | **IR** |
| **PERIOD** |  |  |  |  |  |  |  |  |  |
| **2004-05** | **1.50** | 197.99 | 3 | 262.39 | 3 | 1.14 | 460.38 | 6 | **1.30** |
| **2006-07** | **1.67** | 653.47 | 11 | 881.71 | 6 | 0.68 | 1535.18 | 17 | **1.10** |
| **2008-09** | **1.83** | 1416.79 | 26 | 1364.55 | 9 | 0.66 | 2781.34 | 35 | **1.26** |
| **2010-11** | **2.00** | 1623.55 | 32 | 1083.63 | 3 | 0.28 | 2707.18 | 35 | **1.31** |
| **TOTAL** |  | 3891.79 |  | 3592.28 | 21 |  | 7484.07 |  |  |
|  | **CoRIS - HETEROSEXUALS** | | | | | | | | |
|  | **SIMULATION ANALYSIS**  **(n=850)** | | | **MAIN ANALYSIS**  **(n=621 )** | | | **GLOBALS RESULTS**  **(n=1,471 )** | | |
|  | **IR** | **Person-years** | **# Serocon-verters** | **Person-years** | **# Serocon-verters** | **IR** | **Person-years** | **# Serocon-verters** | **IR** |
| **PERIOD** |  |  |  |  |  |  |  |  |  |
| **2004-05** | **1.50** | 184.51 | 3 | 167.84 | 3 | 1.79 | 352.35 | 6 | **1.64** |
| **2006-07** | **1.67** | 694.84 | 12 | 509.45 | 11 | 2.16 | 1204.29 | 23 | **1.88** |
| **2008-09** | **1.83** | 1293.24 | 24 | 704.01 | 3 | 0.43 | 1997.25 | 27 | **1.34** |
| **2010-11** | **2.00** | 1340.08 | 27 | 465.13 | 2 | 0.43 | 1805.21 | 29 | **1.60** |
| **TOTAL** |  | 3512.67 |  | 1846.42 | 19 |  | 5359.09 |  |  |
