# Supplementary material for: Incidence of Hepatitis C Virus (HCV) in a Multicenter Cohort of HIV-Positive Patients in Spain 2004–2011: Increasing Rates of HCV Diagnosis but Not of HCV Seroconversions
Source: PLoS One. 2014 Dec 30;9(12):e116226. doi: 10.1371/journal.pone.0116226 (PMC4280214; doi:10.1371/journal.pone.0116226)
Supplement: File S3 — Sensitivity analysis excluding the information of HCV serologies performed before cohort entry. - Table 6. Evolution of HCV diagnosis rates and of HCV infection rates (per 100 p-y) - Table 7. Rates and associated risk factors of HCV infection. - Figure 3. Time trends of rates of HCV diagnoses, of HCV infections and of follow-up HCV tests, 2004–2011. (DOC) [file pone.0116226.s003.doc]

**SENSITIVITY ANALYSIS (Excluding the information of HCV serologies performed before cohort entry)**

**Table 6. Evolution of HCV diagnosis rates and of HCV infection rates (per 100 person-years)**

| **All subjects** | | | | | | | | | |
| --- | --- | --- | --- | --- | --- | --- | --- | --- | --- |
|  | **HCV diagnoses (n=1,490 )** | | | **HCV Infections** | | | | | |
|  |  | | | **Midpoint Method (n=1,490)** | | | **Multiple Imputation Method (n=1,490)** | | |
|  | **n** | **IR** | **IRR (95% CI)** | **n** | **IR** | **IRR (95% CI)** | **n** | **IR** | **IRR (95% CI)** |
| **PERIOD** |  |  |  |  |  |  |  |  |  |
| 2004-05 | 3 | 0.99 | 1 | 5 | 1.65 | 1 | 5.85 | 1.90 | 1 |
| 2006-07 | 8 | 0.79 | 0.80 (0.22-2.88) | 10 | 0.99 | 0.60 (0.22-1.63) | 10.20 | 1.00 | 0.53 (0.15-1.88) |
| 2008-09 | 9 | 0.60 | 0.61 (0.09-4.21) | 12 | 0.80 | 0.49 (0.14-1.74) | 8.75 | 0.58 | 0.30 (0.07-1.40) |
| 2010-11 | 13 | 1.10 | 1.11 (0.17-7.09) | 6 | 0.51 | 0.31 (0.09-1.04) | 8.20 | 0.69 | 0.36 (0.08-1.55) |
| **TOTAL** | 33 | 0.82 |  | 33 | 0.83 |  | 33 | 0.83 |  |
|  | **Men who have sex with men** | | | | | | | | |
|  | **HCV diagnoses (n=972 )** | | | **HCV Infections** | | | | | |
|  |  | | | **Midpoint Method (n=972)** | | | **Multiple Imputation Method (n=972)** | | |
|  | **n** | **IR** | **IRR (95% CI)** | **n** | **IR** | **IRR (95% CI)** | **n** | **IR** | **IRR (95% CI)** |
| **PERIOD** |  |  |  |  |  |  |  |  |  |
| 2004-05 | 1 | 0.54 | 1 | 2 | 1.09 | 1 | 1.80 | 0.95 | 1 |
| 2006-07 | 3 | 0.48 | 0.88 (0.08-10.25) | 3 | 0.48 | 0.44 (0.04-4.57) | 4.75 | 0.74 | 0.78 (0.07-8.21) |
| 2008-09 | 5 | 0.54 | 1.00 (0.07-14.5) | 8 | 0.87 | 0.80 (0.17-3.75) | 5.25 | 0.55 | 0.58 (0.07-4.63) |
| 2010-11 | 7 | 0.93 | 1.72 (0.16-18.9) | 3 | 0.40 | 0.37 (0.07-1.86) | 4.20 | 0.55 | 0.58 (0.08-4.08) |
| **TOTAL** | 16 | 0.64 |  | 16 | 0.64 |  | 16.00 | 0.65 |  |
|  | **Heterosexually acquired cases** | | | | | | | | |
|  | **HCV diagnoses (n=461)** | | | **HCV Infections** | | | | | |
|  |  | | | **Midpoint Method (n=461)** | | | **Multiple Imputation Method (n=461)** | | |
|  | **n** | **IR** | **IRR (95% CI)** | **n** | **IR** | **IRR (95% CI)** | **n** | **IR** | **IRR (95% CI)** |
| **PERIOD** |  |  |  |  |  |  |  |  |  |
| 2004-05 | 2 | 1.91 | 1 | 2 | 1.92 | 1 | 3.35 | 3.07 | 1 |
| 2006-07 | 4 | 1.13 | 0.59 (0.11-3.26) | 7 | 1.99 | 1.04 (0.16-6.83) | 5.00 | 1.37 | 0.45 (0.07-2.99) |
| 2008-09 | 4 | 0.77 | 0.40 (0.04-4.40) | 2 | 0.39 | 0.20 (0.02-2.61) | 2.35 | 0.42 | 0.14 (0.01-1.55) |
| 2010-11 | 3 | 0.76 | 0.40 (0.05-3.00) | 2 | 0.51 | 0.26 (0.04-1.56) | 2.30 | 0.57 | 0.19 (0.03-1.06) |
| **TOTAL** | 13 | 0.95 |  | 13 | 0.95 |  | 13.00 | 0.96 |  |

**Table 7: Rates and associated risk factors of HCV infection**

92 %

87 %

25 %

74 %

94 %

92 %

89 %

87 %

29 %

25 %

74 %

|  | ALL subjects (n=1,490) | | | | |
| --- | --- | --- | --- | --- | --- |
|  | **Infections** | **Person-years** | **IR** | **IRR (CI 95%)** | **aIRR (CI 95%)** |
| **SEX** |  |  |  |  |  |
| Male | 24 | 3295.04 | 0.73 | 1 | 1 |
| Female | 9 | 700.97 | 1.28 | 1.76 (0.82-3.80) | 1.71 (0.72-4.06) |
| **TRANSMISSION CATEGORY** |  |  |  |  |  |
| Heterosexuals | 13 | 1369.47 | 0.95 | 1 | 1 |
| Injecting drug users | 3 | 40.14 | 7.47 | 7.87 (1.56-39.8) | 8.67 (1.64-45.7) |
| Homo/bisexual men | 16 | 2486.03 | 0.64 | 0.68 (0.26-1.77) | 1.05 (0.34-3.20) |
| Other /Unknown | 1 | 100.37 | 1.00 | 1.05 (0.14-7.62) | 1.20 (0.16-9.16) |
| **AGE AT ENTRY** |  |  |  |  |  |
| < =30 years | 7 | 1280.21 | 0.55 | 1 | 1 |
| 31-40 years | 14 | 1703.16 | 0.82 | 1.50 (0.52-4.32) | 1.54 (0.53-4.47) |
| 41-50 years | 8 | 658.74 | 1.21 | 2.22 (1.11-4.43) | 1.76 (0.85-3.64) |
| > 50 years | 4 | 353.90 | 1.13 | 2.07 (1.03-4.14) | 1.89 (0.80-4.48) |
| **CD4+ T-Cell Count** |  |  |  |  |  |
| <200 cells/mm^3^ | 7 | 344.63 | 2.03 | 2.83 (1.56-5.15) | 2.33 (1.18-4.61) |
| >= 200 cells/mm^3^ | 26 | 3629.07 | 0.72 | 1 | 1 |
| Not available |  | 22.31 | 0.00 | --- | --- |
| **TOTAL** | 33 | 3996.01 | 0.83 |  |  |
|  | Men who have sex with men (n=972) | | | | |
|  | **Infections** | **Person-years** | **IR** | **IRR (CI 95%)** | **aIRR (CI 95%)** |
| **AGE AT ENTRY** |  |  |  |  |  |
| <=30 years | 3 | 856.10 | 0.35 | 1 | 1 |
| 31-40 years | 7 | 1133.62 | 0.62 | 1.76 (0.35-8.79) | 1.76 (0.35-8.79) |
| 41-50 years | 5 | 328.93 | 1.52 | 4.34 (1.97-9.57) | 4.34 (1.97-9.57) |
| > 50 years | 1 | 167.38 | 0.60 | 1.70 (0.62-4.72) | 1.70 (0.62-4.72) |
| **TOTAL** | 16 | 2486.03 | 0.64 |  |  |
|  | **Heterosexually acquired cases (n=461)** | | | | |
|  | **Infections** | **Person-years** | **IR** | **IRR (CI 95%)** | **aIRR (CI 95%)** |
| **SEX** |  |  |  |  |  |
| Male | 4 | 700.38 | 0.57 | 1 | 1 |
| Female | 9 | 669.10 | 1.35 | 2.36 (0.82-6.77) | 2.57 (0.87-7.55) |
| **CD4+ T-Cell Count** |  |  |  |  |  |
| <200 cells/mm^3^ | 4 | 197.67 | 2.02 | 2.62 (1.04-6.56) | 2.90 (1.13-7.44) |
| >= 200 cells/mm^3^ | 9 | 1164.19 | 0.77 | 1 | 1 |
| Not available | 0 | 7.61 |  | --- | --- |
| **TOTAL** | 13 | 1369.47 | 0.95 |  |  |
| (*) The table shows all the variables included in the final multivariables models | | | | | |

94 %

92 %

89 %

87 %

29 %

25 %

74 %

**Figure 3: Time trends of rates of HCV diagnoses, of HCV infections and of follow-up HCV tests, 2004-2011**

**All Subjects**

Figure 3A

**Men who have sex with men**

Figure 3B

**Heterosexually acquired cases**

Figure 3C
