# Supplementary material for: Incidence of Hepatitis C Virus (HCV) in a Multicenter Cohort of HIV-Positive Patients in Spain 2004–2011: Increasing Rates of HCV Diagnosis but Not of HCV Seroconversions
Source: PLoS One. 2014 Dec 30;9(12):e116226. doi: 10.1371/journal.pone.0116226 (PMC4280214; doi:10.1371/journal.pone.0116226)
Supplement: File S4 — Sensitivity analysis excluding HCV diagnoses that could not be confirmed. - Table 8. Evolution of HCV diagnosis rates and of HCV infection rates (per 100 person-years). - Table 9. Rates and associated risk factors of HCV diagnoses and HCV Infections. - Figure 4. Time trends of rates of HCV diagnoses, of HCV infections and of follow-up HCV tests, 2004–2011. (DOC) [file pone.0116226.s004.doc]

**SENSITIVITY ANALYSES (Excluding HCV diagnoses that could not be confirmed):**

**Table 8. Evolution of HCV diagnosis rates and of HCV infection rates (per 100 person-years)**

| **All subjects** | | | | | | | | | |
| --- | --- | --- | --- | --- | --- | --- | --- | --- | --- |
|  | **HCV diagnoses (n=2,122 )** | | | **HCV Infections** | | | | | |
|  |  | | | **Midpoint Method (n=2,115)** | | | **Multiple Imputation Method (n=2,121)** | | |
|  | **n** | **IR** | **IRR (95% CI)** | **n** | **IR** | **IRR (95% CI)** | **n** | **IR** | **IRR (95% CI)** |
| **PERIOD** |  |  |  |  |  |  |  |  |  |
| 2004-05 | 3 | 0.66 | 1 | 6 | 1.33 | 1 | 6.90 | 1.50 | 1 |
| 2006-07 | 9 | 0.62 | 0.94 (0.28-3.15) | 16 | 1.11 | 0.84 (0.37-1.87) | 13.15 | 0.91 | 0.60 (0.21-1.70) |
| 2008-09 | 16 | 0.74 | 1.12 (0.24-5.30) | 14 | 0.66 | 0.49 (0.17-1.40) | 12.80 | 0.59 | 0.39 (0.12-1.34) |
| 2010-11 | 20 | 1.24 | 1.87 (0.35-10.02) | 5 | 0.31 | 0.24 (0.10-0.57) | 9.00 | 0.55 | 0.37 (0.12-1.16) |
| **TOTAL** | 48 | 0.85 |  | 41 | 0.73 |  | 41.85 | 0.74 |  |
|  | **Men who have sex with men** | | | | | | | | |
|  | **HCV diagnoses (n=1,421 )** | | | **HCV Infections** | | | | | |
|  |  | | | **Midpoint Method (n=1,416)** | | | **Multiple Imputation Method (n=1,421)** | | |
|  | **n** | **IR** | **IRR (95% CI)** | **n** | **IR** | **IRR (95% CI)** | **n** | **IR** | **IRR (95% CI)** |
| **PERIOD** |  |  |  |  |  |  |  |  |  |
| 2004-05 | 1 | 0.38 | 1 | 2 | 0.76 | 1 | 1.90 | 0.72 | 1 |
| 2006-07 | 4 | 0.45 | 1.19 (0.52-2.71) | 5 | 0.57 | 0.74 (0.24-2.32) | 5.75 | 0.63 | 0.88 (0.17-4.51) |
| 2008-09 | 8 | 0.58 | 1.53 (0.52-4.45) | 9 | 0.66 | 0.86 (0.47-1.58) | 6.85 | 0.48 | 0.67 (0.17-2.58) |
| 2010-11 | 11 | 1.01 | 2.65 (0.38-18.35) | 2 | 0.18 | 0.24 (0.04-1.66) | 4.40 | 0.41 | 0.57 (0.08-3.81) |
| **TOTAL** | 24 | 0.67 |  | 18 | 0.50 |  | 18.90 | 0.53 |  |
|  | **Heterosexually acquired cases** | | | | | | | | |
|  | **HCV diagnoses (n=621)** | | | **HCV Infections** | | | | | |
|  |  | | | **Midpoint Method (n=620)** | | | **Multiple Imputation Method (n=621)** | | |
|  | **n** | **IR** | **IRR (95% CI)** | **n** | **IR** | **IRR (95% CI)** | **n** | **IR** | **IRR (95% CI)** |
| **PERIOD** |  |  |  |  |  |  |  |  |  |
| 2004-05 | 2 | 1.19 | 1 | 3 | 1.79 | 1 | 4.20 | 2.45 | 1 |
| 2006-07 | 4 | 0.78 | 0.65 (0.12-3.60) | 10 | 1.96 | 1.10 (0.25-4.79) | 6.65 | 1.27 | 0.52 (0.11-2.36) |
| 2008-09 | 7 | 0.98 | 0.82 (0.08-8.10) | 3 | 0.43 | 0.24 (0.03-1.74) | 4.20 | 0.57 | 0.23 (0.03-1.54) |
| 2010-11 | 6 | 1.28 | 1.07 (0.11-10.23) | 2 | 0.43 | 0.24 (0.10-0.58) | 2.90 | 0.59 | 0.24 (0.06-1.01) |
| **TOTAL** | 19 | 1.02 |  | 18 | 0.98 |  |  |  |  |

**Table 9: Rates and associated risk factors of HCV diagnoses and HCV Infections**

|  | ALL subjects (n=2,115) | | | | |
| --- | --- | --- | --- | --- | --- |
|  | **Infections** | **Person-years** | **IR** | **IRR (CI 95%)** | **aIRR (CI 95%)** |
| **SEX** |  |  |  |  |  |
| Male | 28 | 4687.60 | 0.60 | 1 | 1 |
| Female | 13 | 941.43 | 1.38 | 2.31 (1.12-4.78) | 1.90 (0.88-4.12) |
| **TRANSMISSION CATEGORY** |  |  |  |  |  |
| Heterosexuals | 18 | 1845.90 | 0.98 | 1 | 1 |
| Injecting drug users | 4 | 52.22 | 7.66 | 7.86 (2.18-28.27) | 8.15 (2.18-30.39) |
| Homo/bisexual men | 18 | 3587.22 | 0.50 | 0.51 (0.19-1.14) | 0.83 (0.25-2.74) |
| Other /Unknown | 1 | 143.70 | 0.70 | 0.71 (0.10-5.04) | 0.87 (0.12-6.35) |
| **AGE AT ENTRY** |  |  |  |  |  |
| < =30 years | 10 | 1862.67 | 0.54 | 1 | 1 |
| 31-40 years | 15 | 2348.01 | 0.64 | 1.19 (0.52-2.74) | 1.21 (0.51-2.83) |
| 41-50 years | 12 | 960.44 | 1.25 | 2.33 (1.27-4.25) | 1.88 (0.95-3.72) |
| > 50 years | 4 | 457.91 | 0.87 | 1.63 (0.81-3.27) | 1.47 (0.65-3.30) |
| **CD4+ T-Cell Count** |  |  |  |  |  |
| <200 cells/mm^3^ | 7 | 454.82 | 1.54 | 2.31 (1.18-4.50) | 1.82 (0.89-3.75) |
| >= 200 cells/mm^3^ | 34 | 5095.23 | 0.67 | 1 | 1 |
| Not available |  | 78.99 | 0.00 | --- | --- |
| **TOTAL** | 41 | 5629.03 | 0.73 |  |  |
|  | Men who have sex with men (n=1,416) | | | | |
|  | **Infections** | **Person-years** | **IR** | **IRR (CI 95%)** | **aIRR (CI 95%)** |
| **AGE AT ENTRY** |  |  |  |  |  |
| <=30 years | 4 | 1310.71 | 0.31 | 1 | 1 |
| 31-40 years | 7 | 1561.66 | 0.45 | 1.47 (0.43-4.97) | 1.47 (0.43-4.97) |
| 41-50 years | 6 | 490.79 | 1.22 | 4.01 (1.92-8.35 ) | 4.01 (1.92-8.35 ) |
| > 50 years | 1 | 224.06 | 0.45 | 1.46 (0.39-5.54) | 1.46 (0.39-5.54) |
| **TOTAL** | 18 | 3587.22 | 0.50 |  |  |
|  | **Heterosexually acquired cases (n=620)** | | | | |
|  | **Infections** | **Person-years** | **IR** | **IRR (CI 95%)** | **aIRR (CI 95%)** |
| **SEX** |  |  |  |  |  |
| Male | 5 | 942.95 | 0.53 | 1 | 1 |
| Female | 13 | 902.95 | 1.44 | 2.72 (1.10-6.69) | 2.79 (1.10-7.07) |
| **CD4+ T-Cell Count** |  |  |  |  |  |
| <200 cells/mm^3^ | 4 | 247.36 | 1.62 | 1.81 (0.74-4.42) | 1.94 (0.79-4.74) |
| >= 200 cells/mm^3^ | 14 | 1569.37 | 0.89 | 1 | 1 |
| Not available | 0 | 29.16 | 0.00 | --- | --- |
| **TOTAL** | 18 | 1845.90 | 0.98 |  |  |
| (*) The table shows all the variables included in the final multivariables models | | | | | |

**Figure 4: Time trends of rates of HCV diagnoses, of HCV infections and of follow-up HCV tests, 2004-2011**

**All Subjects**

Figure 4A

**Men who have sex with men**

Figure 4B

**Heterosexually acquired cases**

Figure 4C
